# Interaction between oxytocin receptor DNA methylation and genotype is associated with risk of postpartum depression in women without depression in pregnancy

**DOI:** 10.3389/fgene.2015.00243

**Published:** 2015-07-21

**Authors:** Aleeca F. Bell, C. S. Carter, Colin D. Steer, Jean Golding, John M. Davis, Alana D. Steffen, Leah H. Rubin, Travis S. Lillard, Steven P. Gregory, James C. Harris, Jessica J. Connelly

**Affiliations:** ^1^Department of Women, Children and Family Health Science, College of Nursing, University of Illinois at Chicago, ChicagoIL, USA; ^2^Kinsey Institute and Department of Biology, Indiana University, BloomingtonIN, USA; ^3^Centre for Child and Adolescent Health, School of Social and Community Medicine, University of BristolBristol, UK; ^4^Department of Psychiatry, University of Illinois at Chicago, ChicagoIL, USA; ^5^Department of Psychology, University of Virginia, CharlottesvilleVA, USA; ^6^Department of Psychiatry and Behavioral Sciences, Developmental Neuropsychiatry, Johns Hopkins University School of Medicine, BaltimoreMD, USA

**Keywords:** ALSPAC, OXTR, DNA methylation, oxytocin receptor, postpartum depression, rs53576, oxytocin, epigenetics

## Abstract

Postpartum depression (PPD) affects up to 19% of women, negatively impacting maternal and infant health. Reductions in plasma oxytocin levels have been associated with PPD and heritability studies have established a genetic contribution. Epigenetic regulation of the oxytocin receptor gene (*OXTR)* has been demonstrated and we hypothesized that individual epigenetic variability at *OXTR* may impact the development of PPD and that such variability may be central to predicting risk. This case-control study is nested within the Avon Longitudinal Study of Parents and Children and included 269 cases with PPD and 276 controls matched on age group, parity, and presence or absence of depressive symptoms in pregnancy as assessed by the Edinburgh Postnatal Depression Scale. *OXTR* DNA methylation (CpG site -934) and genotype (rs53576 and rs2254298) were assayed from DNA extracted from blood collected during pregnancy. Conditional logistic regression was used to estimate odds ratios (ORs) and 95% confidence intervals (CIs) for the association of elevated symptoms of PPD with genotype, methylation, and their interaction adjusted for psychosocial factors (*n* = 500). There was evidence of an interaction between rs53576 and methylation in the *OXTR* gene amongst women who did not have depression prenatally but developed PPD *(p* interaction = 0.026, adjusted for covariates, *n* = 257). Those women with GG genotype showed 2.63 greater odds of PPD for every 10% increase in methylation level (95% CI: 1.37, 5.03), whereas methylation was unrelated to PPD amongst “A” carriers (OR = 1.00, 95% CI: 0.58, 1.73). There was no such interaction among women with PPD and prenatal depression. These data indicate that epigenetic variation that decreases expression of *OXTR* in a susceptible genotype may play a contributory role in the etiology of PPD.

## Introduction

Women often experience elevated symptoms of postpartum depression (PPD) with a prevalence up to 19% (Gaynes et al., 2005; O’Hara and McCabe, 2013; Wisner et al., 2013). This can place infants at increased risk for poor behavioral, cognitive, and social development (Beck, 1998; Tronick and Reck, 2009). Various psycho-social stress related risk factors for PPD have been defined (e.g., low social support and adversity) and heritability of PPD has been described (Corwin et al., 2010), suggesting the importance of allowing for such factors in genetic studies. Epigenetic mechanisms, such as DNA methylation, which modify the transcriptional potential of a gene, may yield a mechanistic explanation for variability in stress reactivity. Seminal studies in rodents (Weaver et al., 2004; Murgatroyd et al., 2009) indicate the importance of early life stress in the development of the epigenetic and transcriptional landscape of the genome. These studies highlight the impact of the social environment on the neuroendocrine system and the impact of stressors on development and behavior. The key to our understanding of genetic risk for disorder is to include and consider epigenetic changes that may be already established before onset of the disorder.

Oxytocin has a key role in regulating emotion, social interaction, and stress reactivity (Carter, 1998; Neumann and Landgraf, 2012). It is also central to normal birth, lactation, and mother–infant attachment (Carter, 1998; Feldman, 2012). In addition, reductions in oxytocin measured in plasma (Skrundz et al., 2011; Stuebe et al., 2013) have been associated with PPD. Oxytocin exerts its effect on the cell through interaction with the oxytocin receptor gene (*OXTR)*, a G-protein coupled receptor that upon ligand binding transduces signal to the nucleus (Gimpl and Fahrenholz, 2001). Transcription of the *OXTR* is modulated by DNA methylation of a group of sites located within the first exon of *OXTR* (Kusui et al., 2001; Kumsta et al., 2013) and methylation of these sites is variable in the general population. Methylation of one of these regulatory sites, *OXTR* CpG site -934, has been implicated in autism spectrum disorder (Gregory et al., 2009), callous-unemotional traits (Dadds et al., 2013), and individual variability in social perception (Jack et al., 2012; Puglia et al., 2015) pointing to an important role for the actions of DNA methylation on *OXTR* in behavior. In mice, deletion of *OXTR* leads to deficits in maternal behavior (Takayanagi et al., 2005; Nishimori et al., 2008). Taken together, these data indicate the likely importance of DNA methylation of *OXTR* as a risk factor for PPD.

Review of the postpartum and peripartum depression literature indicates an under representation of PPD genetic studies, particularly those targeting the oxytonergic system (Skalkidou et al., 2012; Figueiredo et al., 2014). Depression, however, has been associated with *OXTR* single nucleotide polymorphisms (SNPs) including SNPs rs53576 (Costa et al., 2009; Riem et al., 2011; Saphire-Bernstein et al., 2011; McQuaid et al., 2013; Thompson et al., 2014) and rs2254298 (Costa et al., 2009; Kawamura et al., 2010; Thompson et al., 2011; Brüne, 2012; Apter-Levy et al., 2013), although these studies report inconsistent findings in regard to these alleles conferring “risk” for depression. The inconsistencies in the reports of association of these SNPs may depend on ethnicity, background, the nature of behaviors involved, social salience (Apter-Levy et al., 2013), social environment (Kawamura et al., 2010), and individual social factors (Tabak, 2013) in published studies (Macdonald, 2012; Szyf, 2013; Tabak, 2013). For example, data indicate that individuals who carry the rs53576_GG genotype (Bradley et al., 2011, 2013; Sturge-Apple et al., 2012; Lucas-Thompson and Holman, 2013; McQuaid et al., 2013; Raby et al., 2013) or the rs2254298_A allele (Thompson et al., 2011; Brüne, 2012) may be more sensitive to their social environment. Importantly, among studies of adult depression McQuaid et al. (2013) report increased depressive symptoms following early life mistreatment in rs53576_GG individuals and Bradley et al. (2011) found increased emotional dysregulation and disordered attachment style with childhood maltreatment in rs53576_GG individuals. These data suggest that the rs53576 GG genotype may be sensitive to adverse life experience, and the methylation status of *OXTR* in this genotype may be important in understanding PPD.

In the present study, we examined the association between genetic/epigenetic variation in the *OXTR* gene (measured in blood during pregnancy) and PPD (defined as elevated symptoms of depression 8 weeks after birth). We took into account whether elevated symptoms of depression were present in pregnancy, as the proposed association between genetic/epigenetic variation and PPD may differ between women with or without depression in pregnancy. Moreover antenatal depression is a known predictor of PPD. We focused on SNPs rs53576 and rs2254298 and concurrently measured DNA methylation of *OXTR* CpG site -934. We hypothesized that as methylation of *OXTR* increased, risk of PPD would increase and that this may occur due to interaction with a specific genotype (SNPs rs53576 and rs2254298).

## Materials and Methods

### The Study Data Collection

This study utilizes data collected in the Avon Longitudinal Study of Parents and Children (ALSPAC), a survey of 14,541 pregnancies in women who resided in the area of Avon, UK, with an expected date of delivery between April 1991 and December 1992. The ALSPAC study enrolled ∼80% of the eligible pregnancies, resulting in a sample that was demographically similar to the UK population (Boyd et al., 2013a; Fraser et al., 2013). The study began in pregnancy, and collected data from self-report questionnaires, biological samples from mothers and offspring, and medical records (Golding et al., 2001; Boyd et al., 2013b; Fraser et al., 2013). Specifically, blood was sampled in individuals during pregnancy (between 7 and 41 weeks gestation) during normal antenatal care medical visits (*M* = 27.15 weeks, SD = 8.31). Please note that the study website contains details of all the data that is available through a fully searchable data dictionary (http://www.bris.ac.uk/alspac/researchers/data-access/data-dictionary).

For detailed description of sample collection please see the study website: http://www.bristol.ac.uk/alspac/researchers/resources-available/

A nested case-control strategy was used to determine the associations between *OXTR* DNA methylation, *OXTR* genotype, and the interaction of methylation and genotype in regard to PPD. The selection of cases and controls was confined to those deliveries for which obstetric data had been previously abstracted; this comprised a non-random group of 8,369 deliveries that were over-weighted with teenage mothers and depressed mothers. From these deliveries we selected women with singleton surviving live-births who had given permission for DNA extraction and analysis. We documented that there was sufficient DNA available; and assured the availability of information on symptoms of depression both at 32 weeks gestation and 8 weeks postpartum. We recorded parity (defined as the number of previous pregnancies resulting in a live- or still-birth), and maternal age at the time of delivery.

The ALSPAC study was designed to collect data on symptoms of depression during pregnancy and postnatally using the Edinburgh Postnatal Depression Scale (EPDS), which has been validated for use both in pregnancy and postnatally. ALSPAC has reported the prevalence of a high EPDS score (>12) of 13.6% at 32 weeks prenatally and 9.2% post-delivery (Evans et al., 2001). For the present study we similarly define PPD as an EPDS score >12 at 8 weeks postpartum (Cox et al., 1987). There were 295 such pregnancies, 133 with ≥400 ng/μl and 162 with ≥200– <400 ng/μl DNA concentration. The cases were only chosen if matched controls were available. To avoid borderline depression (EPDS scores of 11–12), control pregnancies were selected to have an EPDS score <11 at 8 weeks postpartum. For each case, a control was chosen, as far as possible of the same parity, maternal age, and depression status at 32 weeks gestation. No valid controls were available for seven cases and were omitted. Selection preference was given to controls with the highest DNA concentration; thereafter selection focused on the primiparae wherever possible. The final distribution of 288 case/control pairs in the original study design is shown in Supplementary Table S1.

### Measures

Key measures for our analyses include genetic, epigenetic, and depression variables. Our two genetic measures, SNPs rs2254298 and rs53576, both have three levels that represent genotypes GG, AG, and AA, and are examined as dichotomous (GG vs. A allele, with AG and AA combined) due to the small number of AA participants. The epigenetic variable is DNA methylation of *OXTR* CpG site -934, a continuous (%) variable scaled in 10 point units. Depressive symptoms, measured by the EPDS (Cox et al., 1987) at 32 weeks gestation and at 8 weeks postpartum, are treated as binary measures to indicate a high likelihood of depression (>12) and low likelihood of depression (<11). Most studies using cut-offs between 10 and 13 show 80–90% sensitivity and specificity of the EPDS (Myers et al., 2013) to predict clinical depression and earlier studies in the UK have shown that a cut-off of >12 gave the best prediction of clinical depression (Cox et al., 1987). A validation study in the Avon area compared the results of a clinical psychiatric interview with the answers given to the set of EPDS questions – this confirmed the validity of the instrument for use in the study area (Thorpe et al., 1993).

### Potential Confounders

Choice of potential confounders was based on prior ALSPAC findings and the postnatal depression literature. Among the psychosocial variables tested for association with PPD were the following which were devised using details obtained from the self-completion questionnaires filled in by the women during pregnancy: education level achieved, history of psychopathology (other than depression), family adversity (an index consisting of the number of adverse psychosocial factors present), life events occurring during childhood, life events occurring during the second half of pregnancy and/or the first 2 months after the birth, social support received by the woman and the social network available to her. In addition details of mode of delivery (elective or emergency cesarean, forceps or vacuum extraction, normal vaginal) and gestational age were obtained from the medical records.

### The Epigenetic and Genetic Variants

The *OXTR* is located on chromosome 3p25. It spans 19,206 basepairs (GRCh37/hg19 assembly) and contains four exons and three introns. This gene contains a region within the first intron which is subject to epigenetic control via DNA methylation (Kusui et al., 2001) and methylation varies between individuals (Gregory et al., 2009; Jack et al., 2012; Puglia et al., 2015). Within this gene there are also two genetic variants (D’ = 0.64, *r*^2^ = 0.024) that have been investigated more frequently than others: rs53576 and rs2254298, which are both situated in the third intron of the gene. A schematic representation of the gene and its variants is displayed in Supplementary Figure S1. These variants have not been found to have any clear functional impact on the gene although they may be in linkage disequilibrium with a yet unidentified functional SNP.

There are a number of possibilities for the impact of SNP and methylation at the OXTR locus. As stated in the discussion, it is possible that the rs53576 SNP (or one in linkage disequilibrium with it) may alter transcription levels and that in combination with epigenetic modification of OXTR (which would then further decrease expression in a methylation dependent manner), the levels of expression of the gene would decrease below a critical threshold that would then begin to impact the carrier in an alleleXmethylation specific manner. Alternatively, binding of a molecule that regulates DNA methylation in this region may be affected by a polymorphism, which would allow one genotype to modulate DNA methylation and respond to external cues more readily than the other. Interestingly, there is an EBF1 (early B-cell factor 1) binding site adjacent to the methylated region we studied in OXTR (see UCSC genome browser, GRCh37/hg19, chr3: 8,810,503-8,810,738) and SNPs occur within the predicted binding region that are in linkage disequilibrium with rs53576 (Loth et al., 2013). EBF1 has recently been shown to associate with TET2 (Guilhamon et al., 2013), an enzyme in the DNA demethylation cascade that produces 5-hydroxymethylcytosine, another important epigenetic regulatory molecule. Since the presence of methylated cytosine is necessary for TET2 activity to produce 5-hydroxymethylcytosine, it is possible that individuals who display different levels of DNA methylation may have different regulatory potential at this locus. Importantly, EBF1 is a critical factor in epigenetic regulation of both the brain and the blood, thus providing a potential link between relevant epigenetic levels in the two tissues. This molecule has also recently been identified in a genome wide screen for SNPs that are associated with stress (Singh et al., 2014). Clearly, additional research is needed to determine the functional role of the interactions described here.

### Epigenotyping Procedures

The epigenetic variable is DNA methylation of *OXTR* CpG site -934, a continuous (%) variable scaled in 10 point units. Epigenotyping was performed as previously reported (Jack et al., 2012). Two hundred nanograms of DNA extracted from whole blood was subject to bisulfite treatment (Kit MECOV50, Invitrogen, Carlsbad, CA, USA). This converts all non-methylated cytosines in the genome to uracil and allows for the downstream detection of methylated cytosines by sequencing. Ten nanograms of bisulfite converted DNA was used as a template for PCR using a Pyromark PCR kit (Qiagen, Valencia, CA, USA) and 0.2 uM primers TSL101F (5′-TTGAGTTTTGGATTTAGATAATTAAGGATT-3′) and TSL101R (5′-biotin-AATAAAATACCTCCCACTCCTTATT CCTAA-3′). Samples were amplified in triplicate using the following cycling conditions [Step 1: (95°C/15 min)/1 cycle, Step 2: (94°C/30 s, 56°C/30 s, 72°C/30 s)/50 cycles, Step 3: (72°C/10 min)/1 cycle, Step 4: 4°C hold]. This amplifies a region on the coding strand of the *OXTR* gene that contains site -934 (GRCh37/hg19, chr3: 8,810,729-8,810,845). PCR conditions were determined using a set of standards for site -934 at 0, 25, 50, 75, and 100% methylated. Successful PCR amplification of a single fragment that runs at 116 basepairs was confirmed using agarose gel electrophoresis for each sample and replicate. Underlined nucleotides in primer set indicate insertion of an A or C nucleotide at a variable position(C/T) due to a CpG site within the primer. All samples were amplified in triplicate and randomized for pyrosequencing to account for plate and run variability. On average, samples deviated from the mean ± 2.4% (CpG site -934). Pyrosequencing was performed using primer TSL101S (5′-AGAAGTTATTTTATAATTTTT-3′) on a Pyromark Q24 using PyroMark Gold Q24 Reagents (Qiagen, Valencia, CA, USA) per the manufacturer’s protocol. Each pyrosequencing plate contained a set of standards (0, 25, 50, 75, 100% methylated) to evaluate plate-to-plate variability; on average standards deviated from the mean less than or equal to ±0.05%. Epigenotypes reported are an average of three replicates.

### Genotyping Procedures

DNA isolated from whole blood was amplified for each subject by PCR using a Pyromark PCR kit (Qiagen, Valencia, CA, USA), 10 ng of template, and 0.2 uM primers. The primers used for this study and PCR conditions can be found in Supplementary Table S2. Successful PCR amplification of a single fragment that runs at 83 base pairs (rs53576) or 51 basepairs (rs2254298) was confirmed using agarose gel electrophoresis for each sample. Pyrosequencing was performed on a Pyromark Q24 (Qiagen, Valencia, CA, USA) using Pyromark Gold Q24 reagents (Qiagen, Valencia, CA, USA) per the manufacturer’s reagents and protocol. Genotype distribution (and frequency) obtained for rs53576 was as follows: 267 G/Gs (48.8%), 226 A/Gs (41.3%), and 54 A/As (9.9%). This distribution does not deviate from the Hardy–Weinberg equilibrium, χ^2^(1) = 0.369, *p* = 0.543. Genotype distribution (and frequency) obtained for rs2254298 was as follows: 421 G/Gs (77.0%), 116 A/Gs (21.2%), and 10 A/As (1.8%). This distribution does not deviate from the Hardy–Weinberg equilibrium, χ^2^(1) = 0.369, *p* = 0.54. The rs53576 polymorphism was previously genotyped using the Illumina 660W-quad chip on the DNA of the mother. Of the 458 overlapping samples, 455 (99.3%) were identical.

### Statistical Approach

Conditional logistic regression was our primary approach for investigating epigenetic and genetic effects in this case-control PPD design. We examined SNPs (rs2254298 and rs53576) and DNA methylation of CpG site -934 as independent variables, and their interactions, in predicting PPD; while presence or absence of depression in pregnancy was examined as a potential moderator of PPD. Where significant three-way interactions were detected, subgroup analyses were performed in an attempt to simplify the interpretation. Analyses were restricted to white women to avoid genetic confounding due to race (non-White *n* = 18). Models were tested including main effects, interactions, and in the case of three-way interactions, all nested two-way interactions. For adjusted analyses we used five covariates identified using forward and backward stepwise conditional regression on the nine theoretical confounders.

Missing data on genetic variables resulted in two cases being lost in unadjusted analyses. Missing data on confounders led to another 45 cases being lost. This reduced sample size in adjusted analyses resulted in two strata with two cases (depressive symptoms during pregnancy, parity = 1 or 3+, and age 35 and older) not matched to any controls. We combined these uninformative strata with strata having the same depression and parity characteristics, but differing on age group (30–34 years) after assessing for comparable findings. Sensitivity analysis was conducted by comparing the unadjusted models for all available cases with unadjusted models on the reduced sample. Crude and adjusted effects are summarized using odds ratios (ORs), stratified by subgroup, to aid in interpretation. All analyses were performed using SAS 9.3.

### Ethics

Ethical approval for the study was obtained from the ALSPAC Ethics and Law Committee.

## Results

### Sample Characteristics

The characteristics of the mothers analyzed in this study are shown in **Table 1**. In all, 545 mothers of white ethnic origin were available in unadjusted analyses, reducing to 500 in adjusted analyses due to missing data on covariates. Women with and without PPD were equally distributed in two groups per the case-control design. Almost half of the cases had depression defined by EPDS > 12 during pregnancy, and controls were selected to match. Cases had a range in postpartum EPDS scores of 13–27; controls had a range of 0–10. Methylation levels ranged from 17.7 to 70.7%, with no differences in overall averages between cases and controls (Supplementary Figure S2); both SNPs conformed to Hardy–Weinberg equilibrium (see Materials and Methods), the AA genotype was observed for 2% and 10% of the sample for SNPs rs2254298 and rs53576, respectively. The following five confounders were identified as important predictors of case-control status: recent life events, social support, family adversity, childhood events, and poor social network. These were used in adjusted analyses.

**Table 1:** Characteristics of postpartum depression cases and matched controls chosen from the ALSPAC Cohort^a^.

Variable	Description	Control^b^	Case^b^	*p*-value^c^
**Total in analysis (*n* = 545)**				
*N*		276	269	
**Matching criteria**				
Maternal age	<25	71 (25.7)	54 (20.1)	0.3943
	25–29	106 (38.4)	113 (42.0)	
	30–34	77 (27.9)	75 (27.9)	
	35+	22 (8.0)	27 (10.0)	
Parity	0	120 (43.5)	116 (43.1)	0.6404
	1	96 (34.8)	97 (36.1)	
	2	44 (15.9)	35 (13.0)	
	3+	16 (5.8)	21 (7.8)	
Depression in pregnancy^d^	No	141 (51.1)	138 (51.3)	0.9601
	Yes	135 (48.9)	131 (48.7)	
**Genetic information (*n* = 545)**				
*OXTR* rs2254298	GG	211 (76.45)	208 (77.32)	0.8090
	AA/AG	65 (23.55)	61 (22.68)	
*OXTR* rs53576	GG	137 (49.64)	129 (47.96)	0.6945
	AA/AG	139 (50.36)	140 (52.04)	
*OXTR* methylation site -934	%	45.97 [6.80]	46.60 [7.40]	0.2990
**Confounders (*n* = 500)**				
Life events^e^	Score	8.71 [7.41]	13.08 [9.87]	<0.0001
Social support^e^	Score	19.05 [5.11]	16.57 [5.51]	<0.0001
Family adversity^e^	0	63 (25.1)	32 (12.9)	0.0005
	1+	188 (74.9)	217 (87.1)	
Life events in childhood^e^	0–3	76 (30.3)	38 (15.3)	0.0001
	4+	175 (69.7)	211 (84.7)	
Social network^e^	24–29	127 (50.6)	89 (35.7)	0.0008
	3–23	124 (49.4)	160 (64.3)	

*^a^Subsample of Caucasian women.*

*^b^Values are reported as either *n* (%) for categorical variables or mean [SD] for dimensional variables.*

*^c^*p*-values represent the association with case/control status. For categorical variables, the Pearson chi-square test was used while for dimensional variables, Student’s *t*-test were used.*

*^d^EPDS cut-off <11 and >12.*

*^e^Measured in pregnancy.*

### The Effect of OXTR Methylation, Genotype, and their Interaction on PPD

Our primary hypothesis was that increased methylation of *OXTR* enhances risk of PPD in women contingent on *OXTR* genotype (rs53576_GG vs. A allele) and that this might differ according to the presence or absence of depression during pregnancy. Our first modeling goal was to test individual associations between the two specified *OXTR* SNPs (rs53576 and rs2254298) and *OXTR* methylation (CpG site -934) with PPD. As shown in **Table 1**, neither the SNP variables nor methylation were significantly associated with PPD, and these findings were consistent when adjusted for psychosocial covariates (data not shown).

To understand whether PPD was differentially related to methylation, based on genotype and the presence or absence of depression during pregnancy, models with main effects and interactions (methylation × SNP × depression and nested two-way terms) were calculated and simplified to retain only significant terms and nested components. No genetic/epigenetic interactions were found to predict PPD in the total sample (**Table 1**). However we identified a significant three-way interaction between *OXTR* rs53576 genotype, *OXTR* methylation, and depression in pregnancy in both unadjusted (*p* = 0.0192) and adjusted (*p* = 0.0081) models (Supplementary Table S3). The distribution of methylation is shown for each group in Supplementary Figure S3. For rs2254298, no interactions were statistically significant (*p* > 0.10).

Adjusted odds ratios (aOR) for PPD related to *OXTR* methylation and rs53576 genotype are shown in **Table 2** (as well as the corresponding SNP × methylation *p*-values, and exact number of individuals per subgroup) for analyses stratified by absence and presence of depression during pregnancy. See Supplementary Table S4 for a breakdown of each subgroup *n* by genotype. The data show:

**Table 2:** Unadjusted and adjusted^a^ odds ratios and 95% confidence intervals for postpartum depression by *OXTR* rs53576 genotype and *OXTR* methylation site -934, stratified by presence or absence of depression in pregnancy.

	Unadjusted (*N* = 545)			Adjusted (*N* = 500)		
	Odds ratio	95% CI	*p*-value^b^	Odds ratio	95% CI	*p*-value^b^
**No depression in pregnancy**	*n* = 279^c∗^			*n* = 257^d∗^		
Methylation (10% change) by rs53576			0.060		0.026
GG	2.07	(1.18, 3.63)		2.63	(1.37, 5.03)	
A carrier	1.01	(0.62, 1.64)		1.00	(0.58, 1.73)	

**Depression in pregnancy**	*n* = 266^e∗^			*n* = 243^f∗^		
Methylation (10% change) by rs53576			0.172		0.155
GG	0.74	(0.46, 1.19)		0.63	(0.37, 1.09)	
A carrier	1.18	(0.71, 1.98)		1.23	(0.61, 2.08)	

*^a^Adjusted models control for psychosocial covariates: childhood life events, recent life events, family adversity, social support, and social network.*

*^b^*p*-value for interaction within each group.*

*^c^Unadjusted model represents 141 controls and 138 cases with no depression in pregnancy.*

*^d^Adjusted model represents 128 controls and 129 cases with no depression in pregnancy.*

*^e^Unadjusted model represents 135 controls and 131 cases with depression in pregnancy.*

*^f^Adjusted model represents 123 controls and 120 cases with depression in pregnancy.*

*^∗^See Supplementary Table S3 for a breakdown of each subgroup *n* by genotype.*

*The Table shows the odds ratios for PPD for each 10% increase in DNA methylation; *p*-values are for interaction between genotype and methylation level within each prenatal depression division. The overall *p*-values for three way interaction were 0.0192 (*n* = 545) and 0.0081 (*n* = 500) for unadjusted and adjusted interactions respectively. Main effects are not shown due to the two way interactions.*

(i) For the subgroup with no depression in pregnancy (*n* = 257), individuals with the rs53576 genotype GG (*n* = 129) demonstrated a greater odds of PPD [aOR = 2.63 (95% CI: 1.37, 5.03)] for every 10% increase in methylation, whereas for A carriers (*n* = 128), there was no difference in odds of PPD regardless of level of methylation [aOR = 1.00 (95% CI: 0.58, 1.73)]. This is a significant interaction (*p* = 0.026). Moreover, when individuals with a previous history of depression were excluded from the analysis, this interaction remained significant (Supplementary Table S5).
(ii) In women with depression in pregnancy, although methylation and rs53576 had no statistically significant effect on PPD, the trend for A carriers (*n* = 127) was that greater methylation was associated with PPD, while the converse was evident for the GG genotype (*n* = 116; **Table 2**).

## Discussion

This study has shown a significant interaction between the rs53576 genotype, the degree of methylation at CpG -934 in *OXTR*, and the presence of prenatal depression on PPD (*p* = 0.0081). Further detail showed that women who do not display depression in pregnancy, but who harbor the rs53576_GG genotype and display high methylation in *OXTR* are nearly three times as likely to develop PPD, in comparison to women of lower methylation levels or carrying the rs53576 A allele.

Thus findings from this study argue for the integrative use of genetic and epigenetic markers in the oxytocin pathway to better understand and predict risk of psychological disorders in the postnatal period, a critical period for healthy mother–infant interaction. These studies also may shed light on the difficulty of replication of genetic effects seen with phenotypes associated with rs53576 genotype (Bakermans-Kranenburg and van Ijzendoorn, 2014; Connelly et al., 2014). The net result may be to silence expression of *OXTR* to some degree resulting in the mother being less sensitive to the effects of oxytocin. This lower sensitivity to oxytocin may affect the mother’s emotional well-being, bonding with the baby, and coping with the complexities of caring for a newborn baby.

Based on earlier work, we hypothesized that methylation of *OXTR* influences levels of gene expression (Kusui et al., 2001; Gregory et al., 2009), which in turn may have resultant effects on receptor protein levels and receptor sensitivity to an oxytocin signal. The flexibility of rs53576_GG individuals may be rooted in their capacity to modulate expression of this gene through DNA methylation. There are a few examples in the literature at other receptor loci (Philibert et al., 2007; Oertel et al., 2012) suggesting the possibility that the *OXTR* rs53576 polymorphism (or one that is in linkage disequilibrium with it) may regulate gene expression. We hypothesize that the combined effect of polymorphism and methylation may lead to changes in *OXTR* gene expression, although as yet no published data exist to indicate whether the genetic effects at this locus have an impact on transcription. Additional research is warranted to determine the functional role of the interactions described here.

Our findings have implications with respect to understanding whether *OXTR* epigenetic modification could interfere with oxytocin’s protective effect on the perinatal maternal brain (Brunton and Russell, 2008). Early identification of susceptibility might allow clinical vigilance for the possible development of PPD, and targeting of preventative interventions. The biologically at-risk women in this study did not display elevated symptoms of depression in pregnancy, but went on to display an increased risk of PPD after birth. This perinatal transition to motherhood can be a stressful period of changing roles, work obligations, reduced sleep, anticipating childbirth with anxiety, infant feeding difficulties, and trauma related to the actual birth experience. It can also be a rewarding time when the mother derives emotional satisfaction from a close relationship with her baby. Oxytocin nurtures the maternal brain promoting reduced stress, emotional well-being, and healthy mother–infant interaction (Brunton and Russell, 2008). *OXTR* DNA methylation level may reflect reduced OXTR production and reduce responsiveness to the protective benefits of endogenous oxytocin during the perinatal period.

This is the first investigation of *OXTR* as a potential clinical epigenetic/genetic biomarker associated with PPD and further research in a population-based study is required before we can be certain that these results were not derived by chance. There remains the question as to why we did not find an association among women whose depression had started in pregnancy. However, it must be recalled that the controls for this group were, by definition, also depressed prenatally. In this comparison set, the controls were chosen for the cases that were depressed prenatally in order to assess whether there were *OXTR* genetic/DNA methylation relationships among the women who continued to be depressed compared with those whose depression had resolved by 8 weeks postpartum. What this study does not do is compare the women who had prenatal depression with those who did not, regardless of whether she developed PPD or not. It is important, however, to recall that the overall interaction (*p* = 0.0081) included the presence of prenatal depression – thus indicating that the etiology of PPD associated with prenatal depression differs from PPD with no prenatal depression. Thus our findings may have implications for the classification of the onset of mood disorder related to pregnancy. In DSM-5 (American Psychiatric Association, 2013) the depressive disorders specifier is “with perinatal onset.” This specifier refers to depression symptoms with onset during during pregnancy or in the first weeks after delivery. Our data are consistent with a specific risk of postpartum onset of mood disorder in women who were not depressed during pregnancy.

The general effects of oxytocin are to facilitate positive experiences and emotion regulation (Feldman et al., 2012; Carter, 2013). However, individual differences are common, and differential sensitivity to social experiences (whether it be stressful or supportive) is a hallmark of oxytocin’s functions (Bartz et al., 2011). From our results we hypothesize that women with rs53576_GG and higher methylation display such differential sensitivity. Although the present study was not designed to identify interactions with specific life events or other genetic vulnerabilities in the women’s pasts that may have precipitated PPD, it did take account of these features in the analyses so that the final models were independent of psychosocial influences.

There are a number of limitations of this study, these include: (i) the numbers of cases and controls in the study. Even though there was a large sample of pregnant women from which to choose, the number of women with PPD was limited. A way around this would have been to increase the number of controls per case. (ii) For the women who had PPD superimposed on prenatal depression, we chose controls matched for prenatal depression – i.e., controls that recovered from their prenatal depression. This was an important comparison, but it could be argued that other control comparisons including women who did not have prenatal depression might have been equally informative. (iii) Blood samples were used to assay DNA methylation at *OXTR*. Though definitive data that relate blood and brain methylation at CpG site -934 are not available, we have previously shown the utility of this assay in prediction of brain endophenotypes of social perception (Jack et al., 2012; Puglia et al., 2015). Even so, the selected tissue may not accurately reflect changes that occur in the brain during pregnancy. (iv) It is not known if *OXTR* methylation level in the blood changes during pregnancy. A detailed study during pregnancy is warranted to establish the mechanism through which the reported methylation differences may differentially impact the postpartum mother. (v) Lastly, a tag SNP approach coupled with analysis of methylation across the *OXTR* gene would provide a thorough analysis by assaying the over 250 CpG sites in *OXTR*, but our goal was to focus on specific sites identified in the published literature. These limitations raise important issues that can be tested in future studies in other populations.

## Conclusion

Depression is especially detrimental in the first year after birth when the mother’s affect and sensitivity to her infant shapes the level of mother–infant emotional engagement, and subsequently impacts the child’s developmental trajectory (Beck, 1995; Feldman and Eidelman, 2007; Shin et al., 2008). Thus, identification of genetic and epigenetic susceptibility to depression in pregnancy may be one key element in a multidisciplinary approach to reduce the development of PPD and hence the adverse sequelae of depression.

## Author Contributions

Drs AB and JC had full access to all the data in the study and take responsibility for the integrity of the data and the accuracy of the data analysis.

*Study concept and design:* Drs AB, CSC, JC, JD, LR, and JG.

*Acquisition, analysis, or interpretation of data:* All authors.

*Drafting of the manuscript:* Drs AB, CSC, and JC.

*Critical revision of the manuscript for important intellectual content:* All authors.

*Statistical analysis:* Drs JG, CS, LR, and AS.

*Obtained funding:* Drs AB, CSC, and JC.

Administrative, technical, or material support: Dr. JG, SG, and TL.

*Study supervision:* Drs AB and JC.

## Role of the Sponsor

The funding source had no role in the design and conduct of the study; collection, management, analysis, and interpretation of the data; preparation, review, or approval of the manuscript; and decision to submit the manuscript for publication.

## Additional Contributions

We are extremely grateful to all the families who took part in this study, the midwives for their help in recruiting them, and the whole ALSPAC team, which includes interviewers, computer and laboratory technicians, clerical workers, research scientists, volunteers, managers, receptionists, and nurses.

## Conflict of Interest Statement

The authors declare that the research was conducted in the absence of any commercial or financial relationships that could be construed as a potential conflict of interest.
