# Supplementary material for: Interaction between oxytocin receptor DNA methylation and genotype is associated with risk of postpartum depression in women without depression in pregnancy
Source: Front Genet. 2015 Jul 21;6:243. doi: 10.3389/fgene.2015.00243 (PMC4508577; doi:10.3389/fgene.2015.00243)

Figure S1. The oxytocin receptor gene and variants pertinent to this study. A schematic of the oxytocin receptor gene showing its four exons (boxes) and three introns (lines). CpG site -934 lies within the first intron and the genetic variants rs53576 and rs2254298 reside within the third intron. Untranslated regions are shown in grey, the ATG demarks the start of translation. Genomic coordinates are given for cytosine -934 and the gene according to build GRCh38.

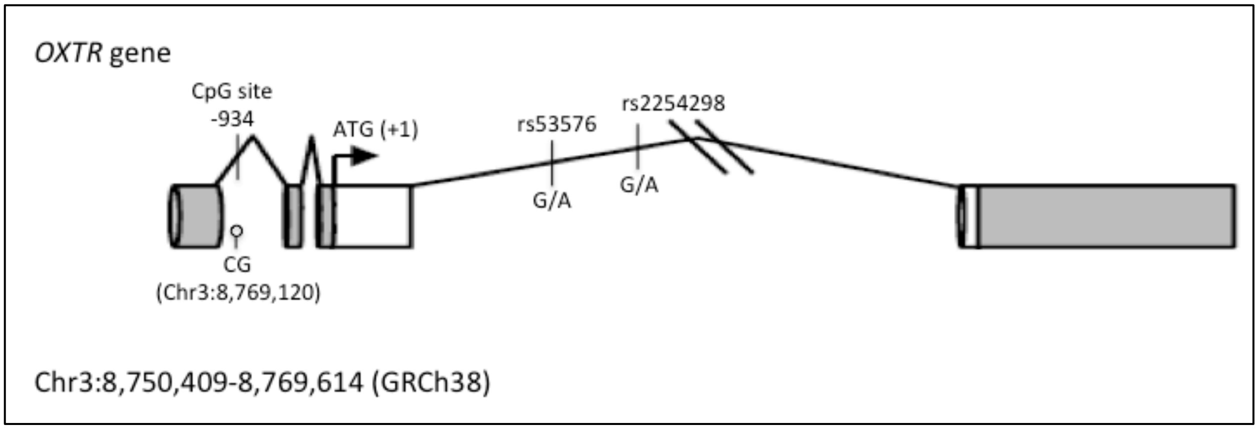

Figure S2. Distribution of *OXTR* methylation in cases and controls. Individual DNA methylation values obtained from each case and control are plotted to show the variability in methylation at this site in our sample. There were no differences in overall averages between cases and controls.

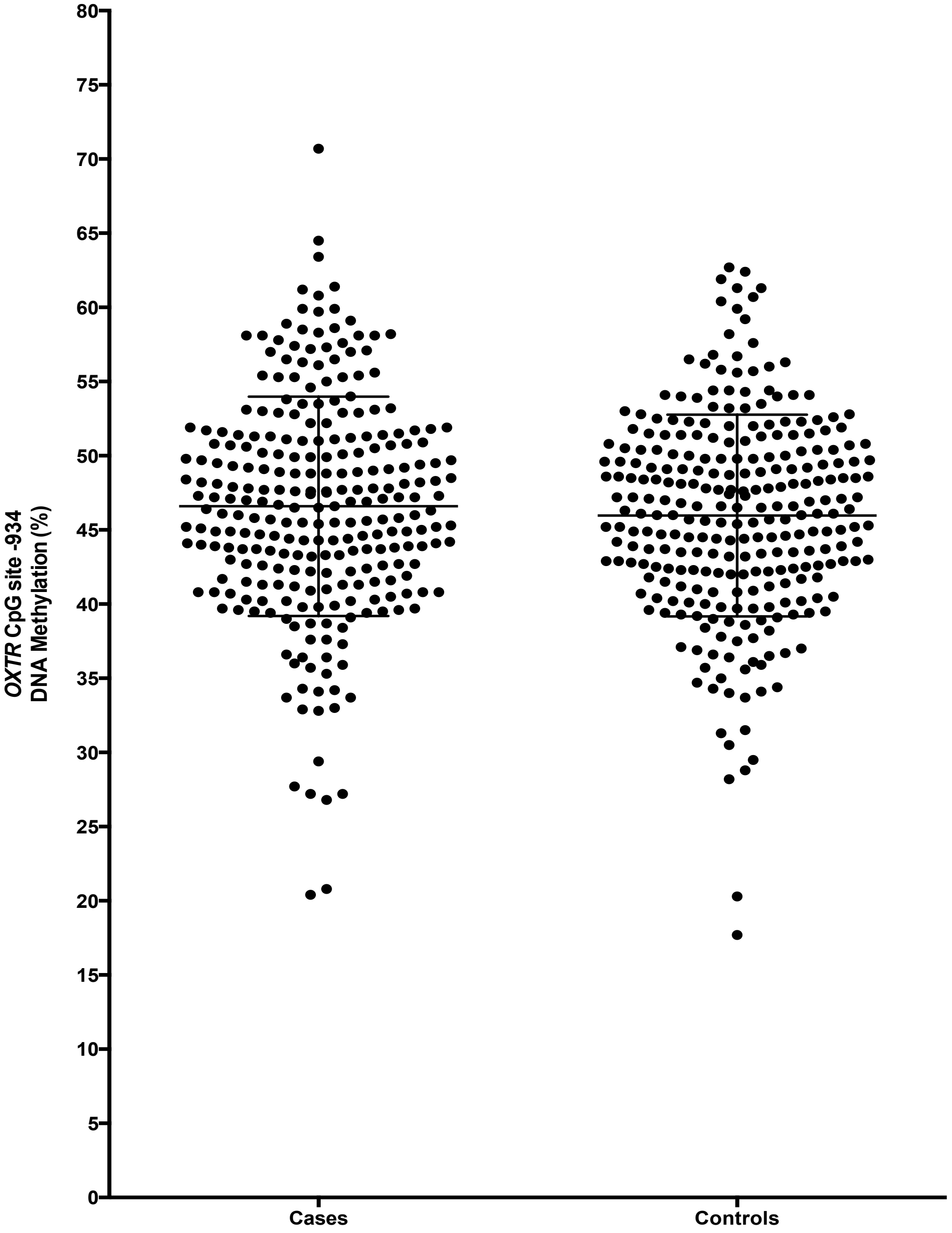

Figure S3. Distribution of methylation in cases and controls by rs53576 genotype. Methylation values for each individual in the study are shown by dotplot for cases and controls by rs53576 genotype. Boxplots display the mean (red bar) and median (black bar) values for each group.

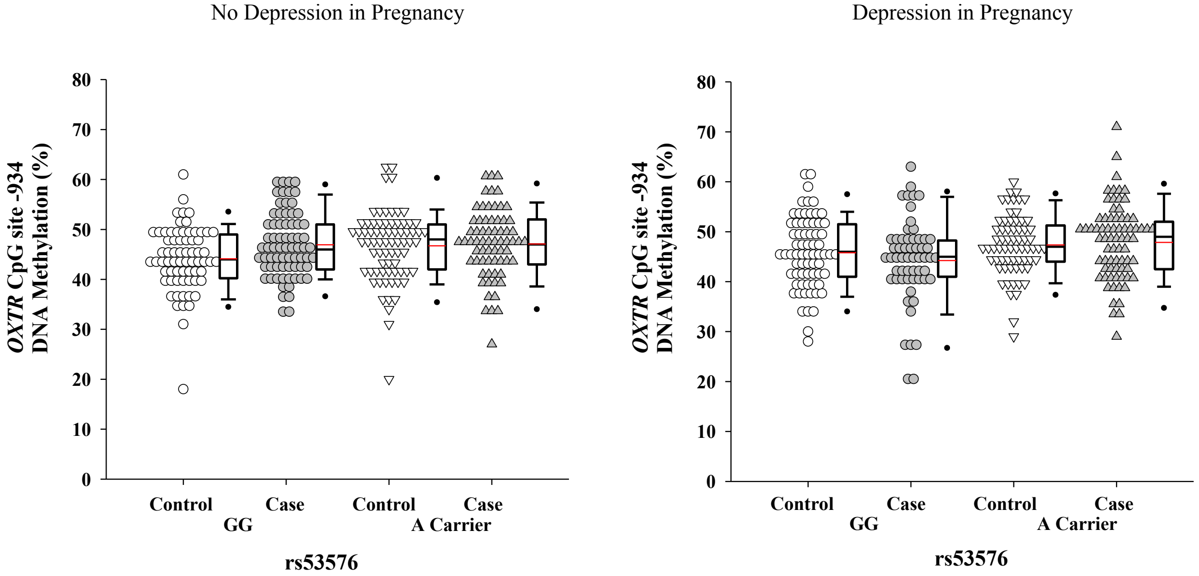

Supplement: Supplementary file 1 [file Image_1.PDF]
