# Supplementary material for: Interaction between oxytocin receptor DNA methylation and genotype is associated with risk of postpartum depression in women without depression in pregnancy
Source: Front Genet. 2015 Jul 21;6:243. doi: 10.3389/fgene.2015.00243 (PMC4508577; doi:10.3389/fgene.2015.00243)
Supplement: Supplementary file 2 [file Table_1.DOC]

**Table S1.** Original Case-Control Design of 288 Pairs of Mothers

|  |  | EPDS at 8 weeks Postpartum | |  |
| --- | --- | --- | --- | --- |
|  |  | Controls | Cases | Total |
| EPDS in Pregnancy | <13 | 146 | 146 | 292 |
|  | 13+ | 142 | 142 | 284 |
|  | TOTAL | 288 | 288 | 576 |
|  |  |  |  |  |
| Parity | 0 | 127 | 127 | 254 |
|  | 1 | 101 | 101 | 202 |
|  | 2 | 44 | 37 | 81 |
|  | 3+ | 16 | 23 | 39 |
|  | TOTAL | 288 | 288 | 576 |
|  |  |  |  |  |
| Maternal Age | <25 | 75 | 65 | 140 |
|  | 25-29 | 111 | 116 | 227 |
|  | 30-34 | 79 | 78 | 157 |
|  | 35+ | 23 | 29 | 52 |
|  | TOTAL | 288 | 288 | 576 |
