# Supplementary material for: Interaction between oxytocin receptor DNA methylation and genotype is associated with risk of postpartum depression in women without depression in pregnancy
Source: Front Genet. 2015 Jul 21;6:243. doi: 10.3389/fgene.2015.00243 (PMC4508577; doi:10.3389/fgene.2015.00243)
Supplement: Supplementary file 3 [file Table_2.DOC]

**Table S2.** Genotyping and sequencing primers used for this study

| rs53576 | Primer sequence | PCR conditions |
| --- | --- | --- |
| TSL102F | 5’-biotin-AAAGGTGTACGGGACATGCC-3’ | Step 1: (95^o^C/15 min)/1 cycle, Step 2: (94^o^C/30 sec, 56^o^C/30 sec, 72^o^C/30 sec)/45 cycles, Step 3: (72^o^C/10 min)/1 cycle, Step 4: 4^o^C hold |
| TSL102R | 5’- TTTCCCCATCTGTAGAATGAGC-3’ |
| TSL102S | 5’-TTCTGTGGGACTGAGG-3’ |
| rs2254298 |  |  |
| TSL103F | 5’-GAAGAAGCCCCGCAAACTG-3’ | Step 1: (95^o^C/15 min)/1 cycle, Step 2: (94^o^C/30 sec, 60^o^C/30 sec, 72^o^C/30 sec)/45 cycles, Step 3: (72^o^C/10 min)/1 cycle, Step 4: 4^o^C hold |
| TSL103R | 5’-biotin-AGTGCCCCTTTCAGGAAACC-3’ |
| TSL103S | 5’-AAGCCCCGCAAACTG-3’ |
