# Supplementary material for: Interaction between oxytocin receptor DNA methylation and genotype is associated with risk of postpartum depression in women without depression in pregnancy
Source: Front Genet. 2015 Jul 21;6:243. doi: 10.3389/fgene.2015.00243 (PMC4508577; doi:10.3389/fgene.2015.00243)
Supplement: Supplementary file 4 [file Table_3.DOCX]

**Table S3.** Case-control status regressed on covariates, genetic and epigenetic main effects, and interactions among genetic, epigenetic, and recent depression indicators in separate models.

|  | Covariates only  (*n*=500) | | Covariates + G/E Main effects (*n*=500) | | Full Model  (*n*=500) | |
| --- | --- | --- | --- | --- | --- | --- |
| Variable | Estimate (SE) | *p*-value | Estimate (SE) | *p*-value | Estimate (SE) | *p*-value |
|  |  |  |  |  |  |  |
| Life Events in Pregnancy | 0.05 (0.01) | 0.0001 | 0.05 (0.01) | 0.0001 | 0.05 (0.01) | <.0001 |
| Social Support | -0.05 (0.02) | 0.0215 | -0.05 (0.02) | 0.0217 | -0.05 (0.02) | 0.0117 |
| Family Adversity | 0.69 (0.30) | 0.0209 | 0.70 (0.30) | 0.0202 | 0.65 (0.30) | 0.0324 |
| Life Events in Childhood | 0.70 (0.24) | 0.0038 | 0.70 (0.24) | 0.0039 | 0.76 (0.25) | 0.002 |
| Social Network | 0.39 (0.21) | 0.0584 | 0.39 (0.21) | 0.0596 | 0.41 (0.21) | 0.0501 |
| rs2254298 GG/A |  | 1.0584 | 0.12 (0.23) | 0.6087 | 0.06 (0.24) | 0.7842 |
| rs53576 GG/A |  | 2.0584 | -0.04 (0.20) | 0.8474 | 4.29 (2.01) | 0.0324 |
| CpG -934 |  | 3.0584 | 0.01 (0.01) | 0.4467 | 0.10 (0.03) | 0.0028 |
| rs53576 GG/A * CpG -934 |  |  |  |  | -0.10 (0.04) | 0.0227 |
| Depression in Pregnancy (Dep in Preg) |  |  |  |  | ^a^ | ^a^ |
| rs53576 GG/A * Dep in Preg |  |  |  |  | -6.77 (2.78) | 0.0148 |
| CpG -934 * Dep in Preg |  |  |  |  | -0.15 (0.04) | 0.0005 |
| rs53576 * CpG -934 * Dep in Preg |  |  |  |  | 0.16 (0.06) | 0.0081 |
|  |  |  |  |  |  |  |
| max-rescaled R^2^ (Nagelkerke) | 0.1825 | | 0.1844 | | 0.2191 | |
