# Supplementary material for: Interaction between oxytocin receptor DNA methylation and genotype is associated with risk of postpartum depression in women without depression in pregnancy
Source: Front Genet. 2015 Jul 21;6:243. doi: 10.3389/fgene.2015.00243 (PMC4508577; doi:10.3389/fgene.2015.00243)
Supplement: Supplementary file 5 [file Table_4.DOCX]

**Table S4.** Sample sizes accounting for genotype and presence or absence of antenatal depression in cases and controls (in unadjusted and adjusted models)

|  |  | Controls  (No PPD) | | Cases  (PPD) | |
| --- | --- | --- | --- | --- | --- |
| *OXTR* rs53576 genotype |  | Unadjusted (*n*=276) | Adjusted  (*n*=251) | Unadjusted (*n*=269) | Adjusted  (*n*=249) |
| AA/AG | Antenatal depression^a^ | 66 | 62 | 73 | 65 |
| AA/AG | No antenatal depression ^b^ | 73 | 67 | 67 | 61 |
| GG | Antenatal^a^ depression | 69 | 61 | 58 | 55 |
| GG | No antenatal depression ^b^ | 68 | 61 | 71 | 68 |

^a^ *n*=243 women in the total sample with antenatal depression

^b^ *n*=257 women in the total sample with no antenatal depression
