# Supplementary material for: Interaction between oxytocin receptor DNA methylation and genotype is associated with risk of postpartum depression in women without depression in pregnancy
Source: Front Genet. 2015 Jul 21;6:243. doi: 10.3389/fgene.2015.00243 (PMC4508577; doi:10.3389/fgene.2015.00243)
Supplement: Supplementary file 6 [file Table_5.DOCX]

**Table S5.** Analysis presented in Table 2 excluding women with a history of depression

|  | Unadjusted  (*n*=429) | | | | | Adjusted  (*n*=409) | | |
| --- | --- | --- | --- | --- | --- | --- | --- | --- |
|  | Odds Ratio | 95% CI | | *p* Value^b^ | | Odds Ratio | 95% CI | *p* Value^b^ |
| **No Depression in Pregnancy** | *n*=246 | | | | | *n*=233 | | |
| Methylation (10% change)  by rs53576 | | | | | 0.030 |  | | 0.026 |
| GG | 2.25 | | (1.24, 4.09) | |  | 2.88 | (1.44, 5.75) |  |
| A Carrier | 0.91 | | (0.53, 1.55) | |  | 1.00 | (0.55, 1.81) |  |
| **Depression in Pregnancy** | *n*=183 | | | | | *n*=176 | | |
| Methylation (10% change)  by rs53576 | | | | | 0.295 |  | | 0.368 |
| GG | 0.73 | | (0.42, 1.27) | |  | 0.68 | (0.36, 1.26) |  |
| A Carrier | 1.13 | | (0.62, 2.06) | |  | 1.06 | (0.49, 2.28) |  |
